# A Fresh Perspective on the Impact of ZnTiO_3_ Coupling on the Microstructure and Photocatalytic Properties of TiO_2_ Fabricated at Varied Temperatures

**DOI:** 10.3390/molecules28227626

**Published:** 2023-11-16

**Authors:** Yuanyuan Zhong, Xiuping Zhang, Yangwen Xia, Ling Zhang, Qiao Xu, Xiaodong Zhu, Wei Feng, Qin Qin

**Affiliations:** 1 School of Mechanical Engineering, Chengdu University, Chengdu 610106, China; zhongyuanyuan@stu.cdu.edu.cn (Y.Z.); zhangxiuping@stu.cdu.edu.cn (X.Z.); x1278704108@163.com (Y.X.); zhangling20030322@163.com (L.Z.); xq13628089297@163.com (Q.X.); 2 Material Corrosion and Protection Key Laboratory of Sichuan Province, Zigong 643002, China; 3 Intelligent Manufacturing College, Chengdu Jincheng College, Chengdu 611731, China; stickerandqq@163.com

**Keywords:** TiO_2_, ZnTiO_3_ coupling, heat treatment temperatures, sol–gel method, photocatalytic activity

## Abstract

ZnTiO_3_/TiO_2_ composite photocatalysts were synthesized via the sol–gel technique, and the impact of varying heat treatment temperatures (470, 570, 670 °C) on their crystalline arrangement, surface morphology, elemental composition, chemical state, specific surface area, optical characteristics, and photocatalytic efficacy was systematically investigated. The outcomes revealed that, as the temperature ascends, pure TiO_2_ undergoes a transition from anatase to rutile, ultimately forming a hybrid crystal structure at 670 °C. The incorporation of ZnTiO_3_ engenders a reduction in the TiO_2_ grain dimensions and retards the anatase-to-rutile phase transition. Consequently, the specimens manifest a composite constitution of anatase and ZnTiO_3_. In contrast, for pure TiO_2_, the specimen subjected to 670 °C annealing demonstrates superior photocatalytic performance due to its amalgamated crystal arrangement. The degradation efficacy of methylene blue (MB) aqueous solution attains 91% within a 60-min interval, with a calculated first-order reaction rate constant of 0.039 min^−1^. Interestingly, the ZnTiO_3_/TiO_2_ composite photocatalysts exhibit diminished photocatalytic activity in comparison to pristine TiO_2_ across all three temperature variations. Elucidation of the photocatalytic mechanism underscores that ZnTiO_3_ coupling augments the generation of photogenerated charge carriers. Nonetheless, concurrently, it undermines the crystalline integrity of the composite, yielding an excess of amorphous constituents that impede the mobility of photoinduced carriers. This dual effect also fosters escalated recombination of photogenerated charges, culminating in diminished quantum efficiency and reduced photocatalytic performance.

## 1. Introduction

With the rapid advancement of industrial technologies, the issue of environmental pollution has become a central concern, drawing attention to the quest for eco-friendly and sustainable energy solutions. One of the most pressing challenges faced by contemporary researchers is the development of such technologies. In contrast to conventional methods for addressing pollution, the use of photocatalytic processes for degrading organic contaminants in wastewater has emerged as a promising and environmentally friendly approach [1,2,3,4,5]. In this context, titanium dioxide (TiO_2_), known for its stable chemical properties, cost-effectiveness, and benign nature, plays a crucial role and finds widespread application in photocatalysis [6,7,8].

The crystalline structure of TiO_2_ is significantly influenced by thermal treatment temperatures. Among the various crystalline phases of TiO_2_ photocatalysts, anatase, and rutile are the most common. Rutile is thermodynamically stable, while anatase gradually transforms into rutile at elevated temperatures [9,10,11]. For instance, Elsellami et al. [11] synthesized TiO_2_ powder using the sol–gel method with titanium tetrachloride (TiCl_4_) as the inorganic precursor, revealing the transition from anatase to rutile within the temperature range of 600–800 °C. Importantly, the convergence of anatase and rutile creates heterophase junctions, resulting in an intrinsic electric field at the interface. This electric field facilitates the separation of photogenerated charges, leading to superior photocatalytic efficiency compared to monophase TiO_2_ counterparts [12,13,14].

Pure TiO_2_ has an intrinsic band gap of 3.2 eV, resulting in limited utilization of solar irradiance, with less than 5% efficiency. Moreover, photogenerated electrons tend to recombine with holes, reducing quantum efficiency and necessitating improvements [15,16,17,18]. By coupling TiO_2_ with other semiconductors, photogenerated electrons can migrate to the semiconductor conduction band with a more positive potential, while holes transfer to the semiconductor valence band with a more negative potential. This promotes the separation of photoinduced electrons and holes [19,20,21,22,23]. A notable study by Sun and colleagues [21] exemplifies this phenomenon, where they fabricated ZnO/TiO_2_ nanocomposites using a simple hydrothermal method, resulting in enhanced charge transfer and improved photocatalytic performance through the formation of heterojunctions.

The impact of semiconductor coupling on the crystal phase behavior of TiO_2_ is a subject of ongoing debate. Numerous studies suggest that semiconductor coupling accelerates the transition from anatase to rutile [24,25]. Conversely, an opposing viewpoint suggests that such coupling hinders the phase transition [26,27]. For instance, Wang et al. [27] synthesized TiO_2_/SiO_2_ photocatalysts through an electrostatic self-assembly method, where SiO_2_ was found to impede the transformation of anatase to rutile.

While ZnTiO_3_/TiO_2_ composite photocatalytic materials have been investigated in several studies [28,29], research on the effects of heat treatment temperatures on the phase transitions and photocatalytic efficiency of ZnTiO_3_/TiO_2_ composites remains limited. This study aimed to comprehensively examine the interplay between calcination temperatures and the microstructural characteristics of both pristine TiO_2_ and ZnTiO_3_/TiO_2_ composite materials. Additionally, using an MB aqueous solution as the target pollutant, the study employed electrochemical measurements, including photocurrent curves and electrochemical impedance spectroscopy (EIS), to elucidate the generation and migration dynamics of photogenerated charges, thereby uncovering the underlying mechanisms of the photocatalytic process. The majority of the literature reports that semiconductor coupling is beneficial for enhancing the photocatalytic performance of materials. In this study, photocurrent tests have demonstrated that the coupling of ZnTiO_3_ leads to the production of more photogenerated electron–hole pairs. However, due to the introduction of ZnTiO_3_, the crystallinity of the sample decreases, resulting in an excess of amorphous components. Electrochemical impedance spectroscopy (EIS) tests reveal that ZT exhibits high resistance to charge carrier migration, limiting the photogenerated electrons and holes that can participate in redox reactions on the particle surface. Photoluminescence (PL) spectroscopy confirms an increased recombination rate of photogenerated electrons and holes in ZT composite materials, leading to a reduction in photocatalytic activity. This finding contradicts previous reports, and the authors arrived at a novel conclusion based on the results of photocatalytic activity experiments and a study of the generation, migration, and recombination of photogenerated charges.

## 2. Results and Discussion

### 2.1. Phase Composition

**Figure 1:**
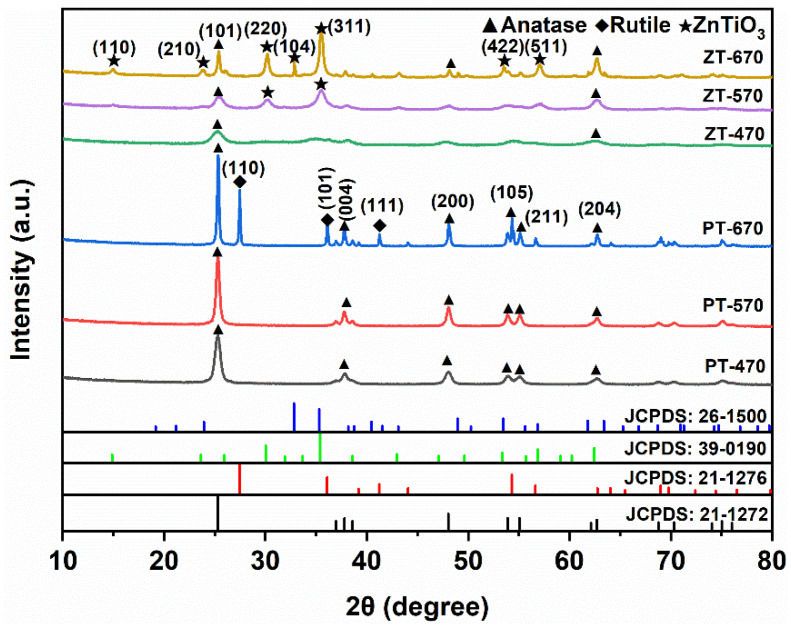
XRD patterns of PT-470, PT-570, PT-670, ZT-470, ZT-570 and ZT-670.

Figure 1 presents XRD patterns of the samples synthesized at different temperatures. When calcinated at 470 °C, clear diffraction peaks are observed at 25.3°, 37.9°, 48.1°, 53.5°, 55.1°, and 62.7°, corresponding to the crystallographic planes (101), (004), (200), (105), (211), and (204) of anatase [JCPDS: 21-1272], consistent with prior studies [30,31]. These distinct peaks confirm the presence of the anatase crystalline structure in both PT and ZT samples. Notably, at 570 °C, the diagnostic peaks associated with anatase become more pronounced, indicating enhanced crystallization. Importantly, no peaks indicative of rutile are observed at this temperature.

At 670 °C, discernible diffraction peaks appear in PT at 27.4°, 36.1°, and 41.2°, corresponding to the crystallographic planes (110), (101), and (111) characteristic of rutile [JCPDS: 21-1276]. This shift suggests the beginning of a phase transition from anatase to rutile, resulting in a coexistence of anatase and rutile in the crystal structure. In the case of ZT-670, the relative intensity of anatase peaks decreases, but no distinct rutile peaks are observed. Simultaneously, diffraction peaks at 30.1°, 32.8°, and 35.5°correspond to the crystallographic planes (220), (104), and (311) of ZnTiO_3_ [JCPDS: 39-0190, JCPDS: 26-1500], confirming the formation of ZnTiO_3_/TiO_2_ composite materials.

This XRD analysis provides insight into the temperature-dependent interplay between anatase and rutile phases in PT and ZT samples while revealing the emergence of composite ZnTiO_3_/TiO_2_ structures at specific calcination temperatures. Understanding these crystalline dynamics enhances our comprehension of the evolving microstructure of the synthesized materials and sets the stage for a comprehensive assessment of their photocatalytic performance.

The Debye–Scherrer [32] Formula (1) is used to calculate the average grain size of TiO_2_:

D = Kλ/βcosθ(1)

where K is a constant of 0.89, λ is the X-ray incident wavelength (0.15418 nm for the Cu target), β is the full width at half maxima (FWHM) of diffraction peak, and 2θ is the Bragg angle.

The relative mass content of anatase (X_A_) in mixed crystal TiO_2_ can be calculated by Formula (2):

X_A_ = (1 + 1.26(I_R_/I_A_))^−1^ (2)

where I_A_ and I_R_ represent the intensities of the anatase (101) plane and rutile (110) plane, respectively.

The results average grain size and mass content of TiO_2_ are shown in Table 1. It can be seen that the grain size increases with the higher temperature. The formation of ZnTiO_3_ refines the grain size and inhibits the transformation of anatase to rutile.

### 2.2. Morphology and BET Surface Area

**Figure 2:**
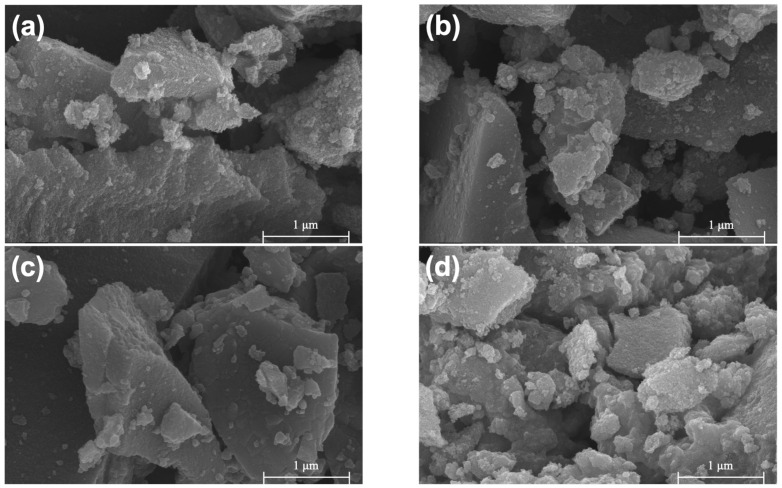

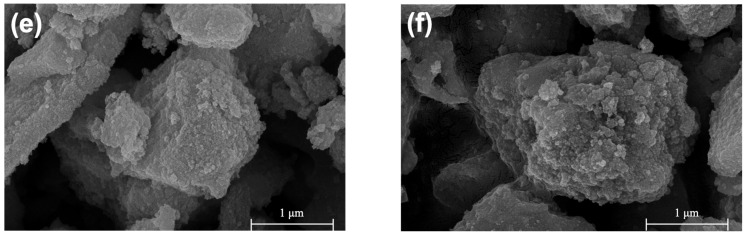
SEM images of PT-470 (**a**), PT-570 (**b**), PT-670 (**c**), ZT-470 (**d**), ZT-570 (**e**), ZT-670 (**f**).

Figure 2 presents scanning electron microscopy (SEM) images of PT (a–c) and ZT (d–f), revealing particles at various stages of aggregation. These aggregates display a wide range of shapes and sizes, spanning from the nanoscale to a few micrometers.

**Figure 3:**
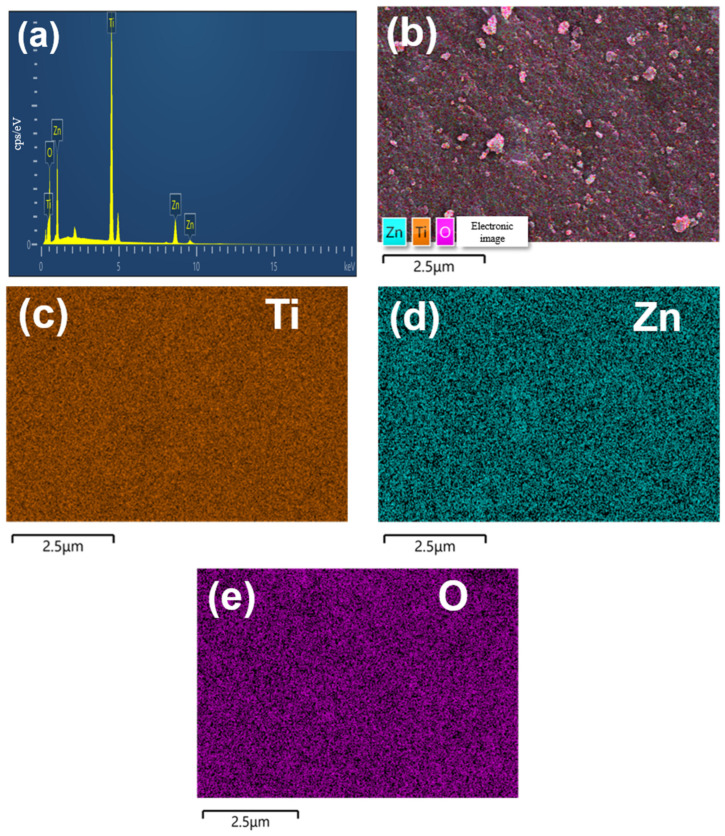
EDS results of ZT-670 (**a**) and element mappings of Ti, Zn, O elements (**b**–**e**).

In Figure 3, elemental composition insights are provided through energy-dispersive X-ray spectroscopy (EDS) results (a) and elemental mappings (b–e) of ZT-670. The composite clearly shows the presence of Zn, Ti, and O elements, confirming the coexistence of Zn within ZT-670. These elemental constituents are uniformly distributed throughout the matrix.

**Figure 4:**
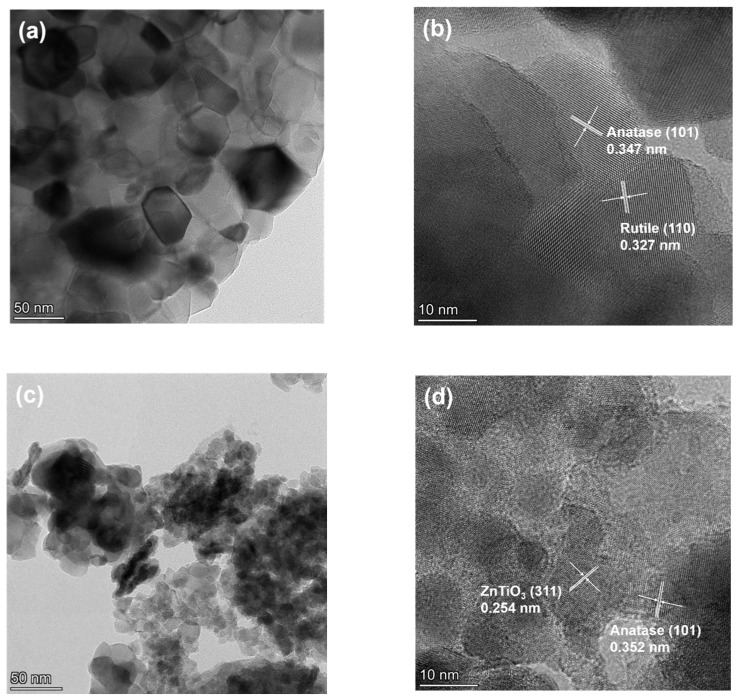
TEM and HRTEM images of PT-670 (**a**,**b**) and ZT-670 (**c**,**d**).

In Figure 4, we delve deeper into the internal structure with TEM and HRTEM images of PT-670 (a,b) and ZT-670 (c,d). These images reveal that the individual particle size of PT is larger than that of ZT, and the lattice fringes are distinctly visible in Figure 4b,d, indicating the materials’ high crystalline quality. Notably, the lattice fringes in Figure 4b,d, measuring 0.347 nm and 0.327 nm, respectively, correspond to the (101) crystal plane of anatase and the (110) crystal plane of rutile [33,34]. This observation corroborates the mixed crystal composition of anatase and rutile in PT-670, consistent with the XRD findings. Furthermore, the spacings of 0.254 nm and 0.352 nm marked in Figure 4d correspond, respectively, to the (311) crystal plane of ZnTiO_3_ [35] and the (101) crystal plane of anatase, confirming the multifaceted composition of ZT-670.

**Figure 5:**
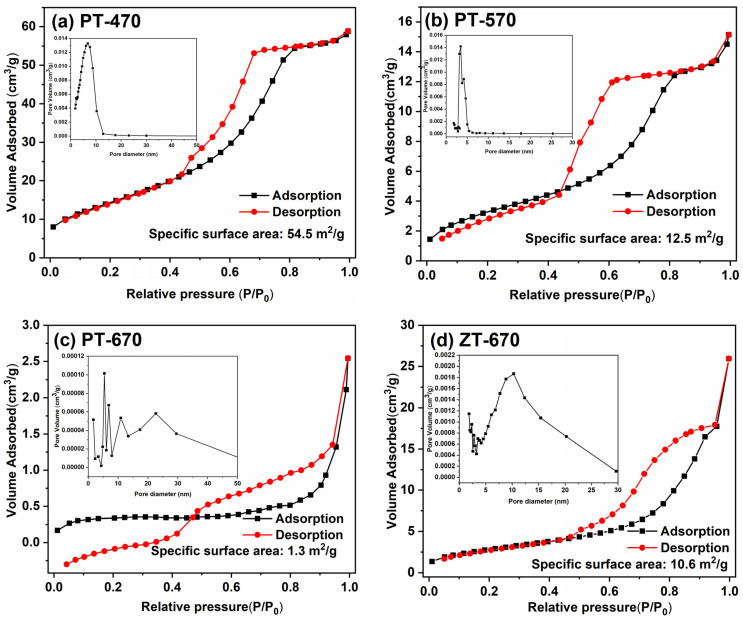
N_2_ adsorption–desorption isotherms of PT-470 (**a**), PT-570 (**b**), PT-670 (**c**), ZT-670 (**d**).

The specific surface area of TiO_2_ materials is of paramount importance in determining their photocatalytic efficacy, as it governs both adsorption capacity and the abundance of catalytically active sites. Figure 5 presents nitrogen adsorption–desorption isotherms, revealing a decreasing trend in specific surface area for PT with successive calcination at 470 °C, 570 °C, and 670 °C, resulting in values of 54.5 m^2^/g, 12.5 m^2^/g, and 1.3 m^2^/g, the specific surface area of samples is shown in Table 2. Notably, this trend highlights the inverse relationship between temperature and specific surface area. The increase in temperature leads to a sharp decrease in surface area, which is due to grain growth and increased agglomeration between particles at high temperatures. When coupled with ZnTiO_3_, the resulting ZT-670 exhibits a specific surface area of 10.6 m^2^/g, indicating the favorable impact of ZnTiO_3_ coupling on surface area.

### 2.3. Element Valence State

**Figure 6:**
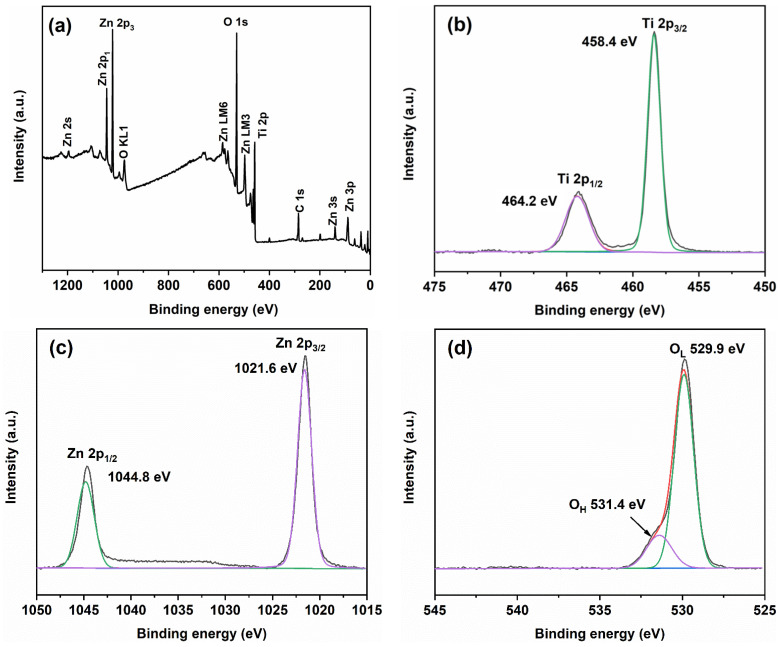
XPS spectrum of ZT-670: (**a**) total spectrum; (**b**) Ti 2p; (**c**) Zn 2p; (**d**) O 1s.

To gain insights into the elemental composition and chemical valence states, XPS measurements were conducted on ZT-670, and the resulting findings are presented in Figure 6. The composite spectra (Figure 6a) prominently exhibit discernible peaks corresponding to C 1s, Ti 2p, O 1s, and Zn 2p, conclusively confirming the presence of Zn within the sample. Taking a closer look, Figure 6b provides a high-resolution depiction of the Ti 2p spectrum, which appears as a bifurcated profile with binding energies of 458.4 eV and 464.2 eV for Ti 2p_3/2_ and Ti 2p_1/2_, respectively. These binding energies unequivocally indicate the presence of Ti in a 4+ valence state, consistent with previous investigations [8,36]. Figure 6c presents the high-resolution spectrum of Zn 2p, where characteristic peaks are observed at 1021.6 eV and 1044.8 eV, corresponding to Zn 2p_3/2_ and Zn 2p_1/2_, respectively. These features unambiguously indicate a valence state of 2+ for the Zn element within the sample, in agreement with prior research [28,29]. The oxygen species are also examined, with Figure 6d elucidating O 1s peaks located at 529.9 eV and 531.4 eV. These distinct energy levels correspond to lattice oxygen and surface hydroxyl groups [29,37], respectively.

### 2.4. Optical Property

**Figure 7:**
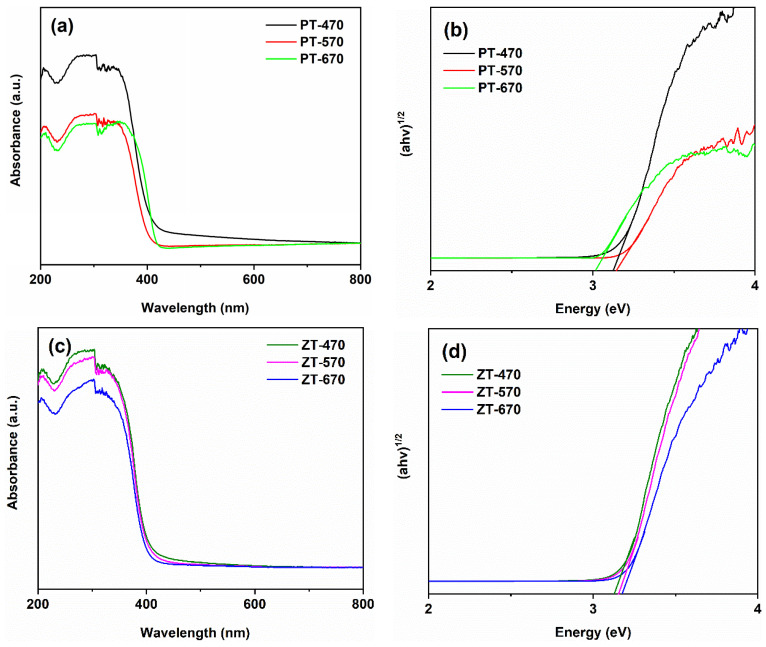
UV-visible absorption spectra (**a**,**c**) and band gaps (**b**,**d**) of PT and ZT.

In Figure 7a, we present the UV-visible absorption spectra of the samples at different temperature levels. Within the PT samples, a clear trend emerges: the absorption threshold of PT-670 exceeds that of PT-470 and PT-570. This observation is further confirmed in Figure 7b, where the band gap of PT-670 is determined to be 3.06 eV, while the values for PT-470 and PT-570 are 3.20 eV and 3.22 eV, respectively. This phenomenon can be attributed to the transformation of a significant fraction (43.7%) of anatase into rutile upon calcination at 670 °C. The narrower band gap of rutile compared to anatase contributes to the reduction in the band gap width of PT-670 relative to PT-470 and PT-570. Focusing on the ZT samples, a remarkable consistency in absorption behavior is observed. The corresponding band gap values are systematically outlined in Table 3. Notably, the coupling of ZnTiO_3_ exhibits an intriguing influence on the phase transition dynamics. This influence is particularly evident in the band gap values, which approach the theoretical anatase value of 3.20 eV [38,39]. This finding underscores the crucial role of ZnTiO_3_ coupling in restraining the conversion of anatase to rutile.

**Figure 8:**
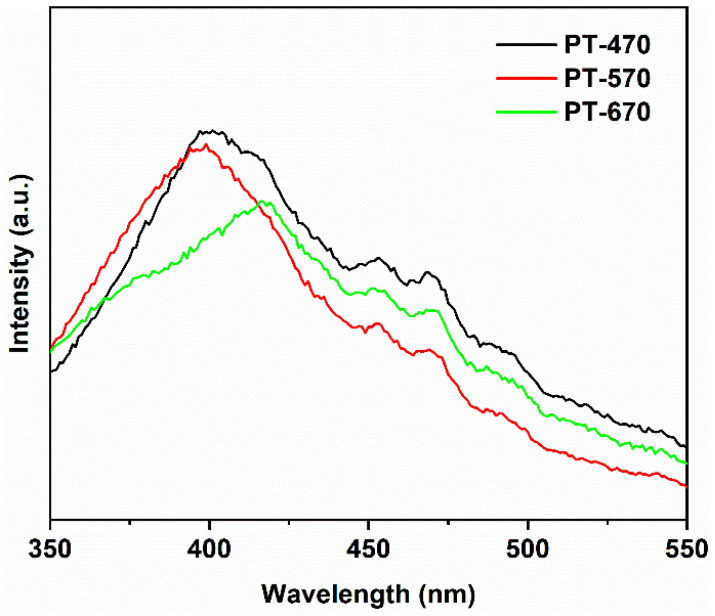
Photoluminescence spectra of PT at different temperatures.

Moving to Figure 8, we delve into the PL spectra of PT samples. Due to energy dissipation, a Stokes shift occurs, resulting in a red shift of approximately 13 nm for the emitted photon [40]. With a band gap of 3.20 eV for PT-470, the main peak is theoretically expected to be around (1240/3.20 + 13) = 400 nm [41,42]. Remarkably, the PL spectra align with this prediction, with the principal peak of PT-470 indeed located near 400 nm. Peaks between 450–470 nm originate from the recombination of photogenerated electrons and holes due to oxygen vacancies and surface defects [43,44]. This resonance validates the theoretical projection. Importantly, the intensity of PL peaks serves as an indicator of photogenerated electron–hole recombination. Among the PT samples, PT-470 exhibits the most pronounced peak intensity, indicative of facile carrier recombination. This can be attributed to recombination centers arising from crystal defects induced by low-temperature calcination. As the temperature increases to 570 °C, improved crystallinity comes into play, reducing charge carrier recombination and resulting in reduced peak intensity. Strikingly, PT-670 records the lowest peak intensity, attributed to two factors: elevated temperature, which enhances crystallinity and reduces defect density and recombination centers, and the coexistence of anatase and rutile in PT-670, with their distinct band potential differences, which enhances charge transfer dynamics, further suppressing recombination [45]. Consistent with the PT-670 band gap of 3.06 eV, the main peak’s expected position aligns with (1240/3.06 + 13) = 418 nm, in agreement with the PL findings.

**Figure 9:**
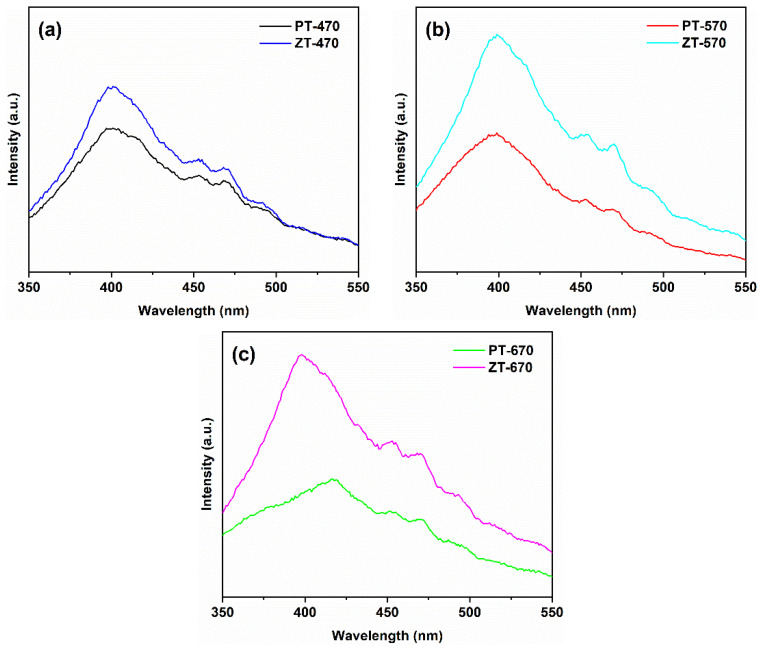
Photoluminescence spectra of PT and ZT at 470 °C (**a**), 570 °C (**b**), 670 °C (**c**).

In Figure 9, we compare the PL spectra of ZT and PT samples at equivalent temperatures. Surprisingly, ZT samples exhibited stronger PL peak intensities than their PT counterparts across all temperatures. This intriguing result suggests that ZnTiO_3_ coupling may not facilitate charge separation but, instead, reduces quantum efficiency. Comparing XRD patterns, the significantly reduced diffraction peak intensity in ZT at equivalent temperatures implies lower crystallinity compared to PT. This lower crystallinity results in a greater number of crystal defects, creating recombination centers for photogenerated charges and amplifying PL peak intensity while concurrently lowering quantum efficiency [46]. The comprehensive analysis of UV-visible absorption and photoluminescence spectra presented in these figures provides a comprehensive view of the optoelectronic characteristics of the synthesized materials. The intricate interplay between phase composition, temperature, and coupling effects emerges as a crucial factor governing their optical behavior.

### 2.5. Photocatalytic Performance

**Figure 10:**
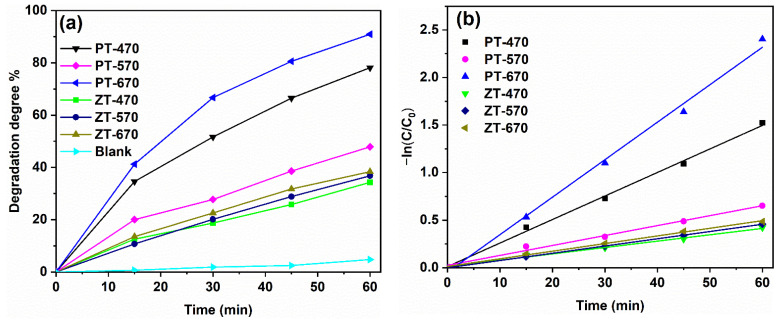
Photodegradation curves (**a**) and kinetics fitting curves (**b**) of samples.

The results of the photocatalytic experiments are presented in Figure 10. After 60 min of illumination, the self-decomposition rate of MB is 4%, indicating that it is not the primary factor contributing to the degradation of MB. Notably, PT-670 stands out as the most efficient performer in terms of photocatalytic activity, achieving an impressive 91% degradation degree within 60 min of reaction time. In stark contrast, PT-470 and PT-570 exhibit lower degradation rates of 78% and 48%, respectively. This achievement underscores the enhanced photocatalytic efficacy brought about by the mixed crystal TiO_2_ structure compared to its monophase counterpart. The coexistence of anatase and rutile within the mixed crystal structure proves instrumental in reducing the recombination of photoinduced charges. As a result, an improvement in quantum efficiency and overall photocatalytic performance is observed. On the contrary, the degradation rates of the ZT samples significantly lag behind those of PT at all three temperatures. The nuanced interplay becomes further elucidated through PL spectra, which highlight the consequences of ZnTiO_3_ coupling on crystallinity. The coupling effect leads to a substantial decline in crystallinity, catalyzing an overabundance of crystal defects and the formation of new recombination centers. This situation is not conducive to the effective utilization of photogenerated charges, resulting in a noticeable reduction in photocatalytic activity [47].

The surface degradation of the photocatalyst follows the Langmuir–Hinshelwood first-order reaction model, expressed as −ln(C/C_0_) = kt, where C_0_ is the initial solute concentration, C is the solute concentration at time t, k is the first-order reaction rate constant, and t is the reaction time [48,49]. The kinetics fitting curves are vividly depicted in Figure 10b, with the resulting k values and R^2^ (linear fitting degree) meticulously documented in Table 4. With R^2^ values approximating 1, it is clear that the photodegradation of MB by the samples conforms to a first-order reaction. Higher k values correspond to faster reaction rates. The collective insights pinpoint that, for PT, the sample calcinated at 670 °C exhibits the most rapid reaction rate. In the context of ZT samples, their reaction rates lag behind those of PT across all three temperatures, corroborating the results derived from photocatalytic degradation experiments. This thorough analysis encapsulates not only the experimentally observed photocatalytic performances but also dissects the kinetics of degradation, corroborating the pivotal role of mixed crystal structures and the adverse effects of ZnTiO_3_ coupling on the photocatalytic behavior of the synthesized materials.

**Figure 11:**
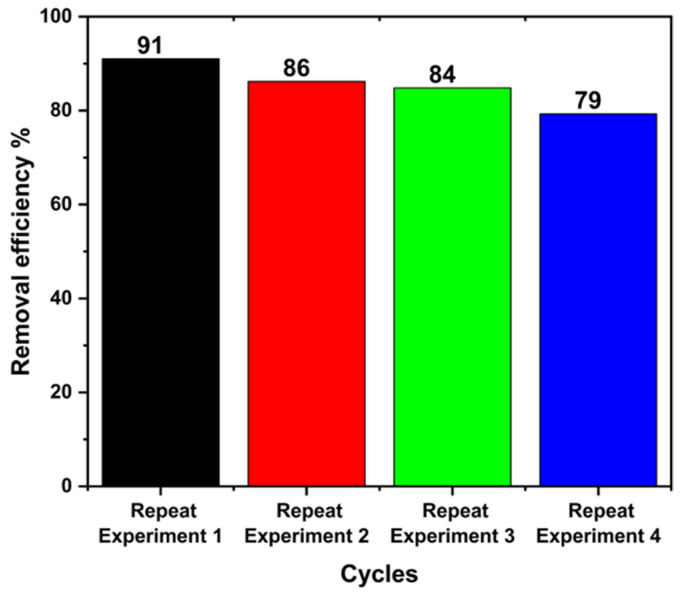
The cyclic experiment of PT-670 photocatalyst for MB degradation.

A cyclic experiment was performed on the PT-670 sample, and the results are shown in Figure 11. After four cycles, the degradation degree was 79%, demonstrating good repeatability.

**Figure 12:**
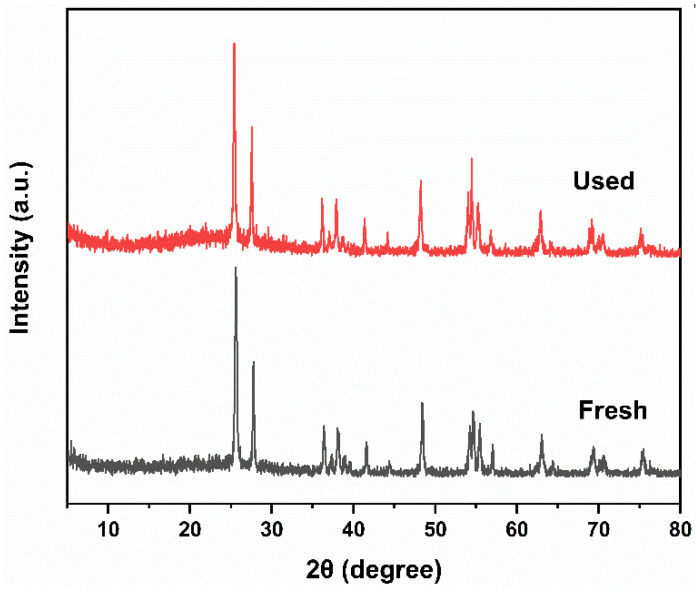
XRD patterns of PT-670 before and after cyclic experiment.

The XRD pattern of PT-670 before and after the cyclic experiment is shown in Figure 12. It can be observed that the intensity and position of the characteristic peaks of the sample remain unchanged compared to before the cyclic experiment, indicating that the prepared sample has good structural stability. The decrease in photocatalytic activity may be caused by the adsorption of the MB molecules on the PT-670 surface [50,51].

**Figure 13:**
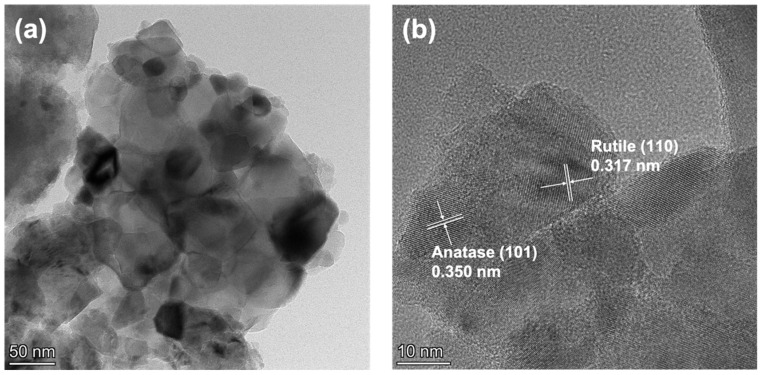
TEM (**a**) and HRTEM (**b**) images of PT-670 after repeat experiment.

The PT-670 sample, subjected to repeated testing, underwent TEM analysis, as illustrated in Figure 13. The particle morphology and dimensions displayed minimal alteration compared to the initial samples. The interplanar distances were measured at 0.350 nm and 0.317 nm, corresponding respectively to the (101) crystal plane of anatase and the (110) crystal plane of rutile. The results demonstrate that, following repeated utilization, the sample still maintained a mixed-crystal structure of anatase and rutile, consistent with the original sample.

The authors summarized several TiO_2_-based photocatalytic materials reported by the literature, as shown in Table 5, indicating that the PT-670 prepared in this study had relatively high activity.

### 2.6. Photocatalytic Mechanism

**Figure 14:**
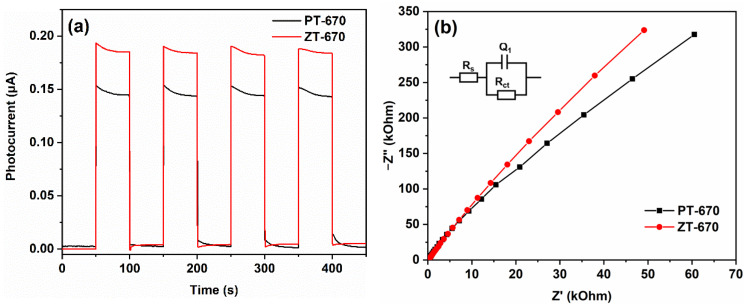
Photocurrent curves (**a**) and electrochemical impedance spectroscopy (**b**) of PT-670 and ZT-670.

Based on the results derived from the photocatalytic experiments, a distinct observation emerges: the photocatalytic performance of ZnTiO_3_/TiO_2_ composites experiences a decline, which diverges from the trends reported in previous studies involving TiO_2_ semiconductor coupling [19,20]. For instance, Vijayan et al. [19] created g-C_3_N_4_/TiO_2_ through a co-precipitation and thermal polymerization approach, where the formation of a heterostructure enhanced the transfer and separation of charge carriers, ultimately leading to increased photocatalytic activity. The essence of the photocatalytic process lies in the generation, separation, and migration of photogenerated charges. Consequently, to delve deeper into the causes underlying the observed decline in photocatalytic performance, it becomes imperative to supplement the insights gained from PL spectra with electrochemical measurements. In pursuit of this clarification, photocurrent curves (a) and EIS Nyquist plots (b) were undertaken, with the results illustrated in Figure 14. When illuminated by photons exceeding the band gap threshold, photocatalysts undergo electron excitation from the valence band to the conduction band, generating photogenerated charges. The subsequent directional movement of these charges manifests as photocurrent. The density of this photocurrent essentially serves as a metric reflecting the capacity of the photocatalyst for charge generation [56]. In this context, the photocurrent density of ZT-670 proves superior to that of PT-670 (Figure 14a), implying a greater yield of photogenerated charges following ZnTiO_3_ coupling. Simultaneously, the ability for photogenerated charge transfer is evaluated through EIS analysis. In accordance with Nyquist’s principle, a larger impedance spectra radius corresponds to increased resistance in charge migration. Conversely, a smaller radius indicates more efficient charge transfer [57]. Notably, the Nyquist diameter of ZT-670 exceeds that of PT-670 (Figure 14b), indicating increased resistance to photogenerated charge migration within ZT-670. This resistance hinders the efficient migration of photogenerated charges, ultimately impeding charge transfer. The increased PL peak intensity in ZT relative to PT corroborates this observation, reaffirming enhanced recombination of photogenerated charges within ZnTiO_3_/TiO_2_ composites.

**Figure 15:**
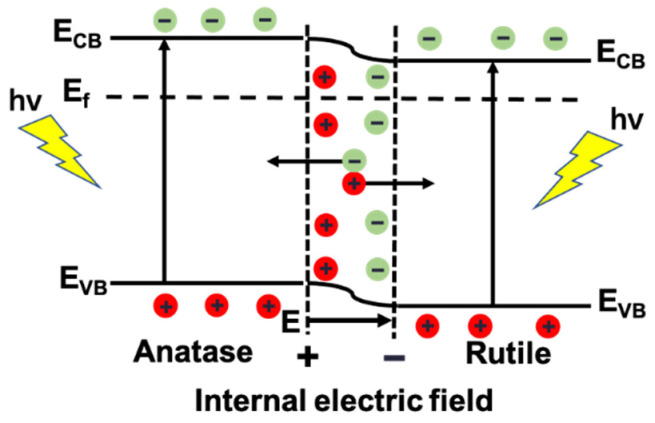
Charge transfer mechanism diagram of PT-670.

The charge transfer mechanism of PT-670 is proposed, as shown in Figure 15. When anatase and rutile contact to form interfaces, electrons near the anatase interface diffuse to rutile, leaving positive charges. The rutile holes spread toward the anatase, leaving negative charges. This diffusion causes the band bending at the interface, creating a layer of space charges, and an electric field that is built in from anatase to rutile [58,59]. The diffusion causes the Fermi levels of the two to converge, resulting in a positive band shift (downward) for anatase and a negative band shift (upward) for rutile. When stimulated by photons, anatase, and rutile both generate photogenerated electrons and holes. As photogenerated charges migrate to the space charge layer at the interfaces, due to the presence of a built-in electric field, electrons transfer to anatase and holes flow to rutile, effectively separating photogenerated charges and improving quantum efficiency [60,61].

**Figure 16:**
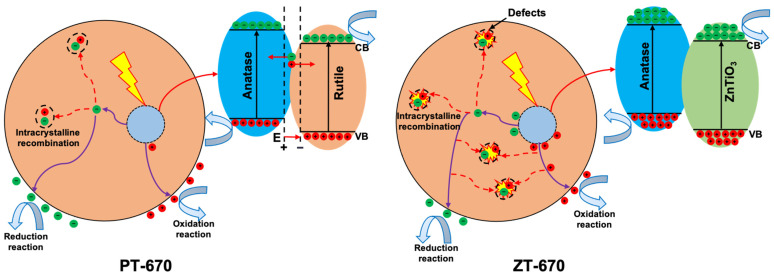
The photogenerated charge generation, migration, and reaction mechanism diagram of ZT-670.

The photogenerated charge generation, migration, and reaction mechanism diagram for ZT-670, is shown in Figure 16. In comparison to PT, the coupling of ZnTiO_3_ with TiO_2_ to form a composite material is conducive to generating more photogenerated electrons and holes. However, during the process of photogenerated charge migration to the particle surface, the reduced crystallinity of the composite material leads to the formation of more crystal defects. These defects serve as recombination centers for photogenerated electrons and holes. Consequently, there are fewer electrons and holes available for oxidation reduction reactions occurring at the particle surface compared to PT-670. As a result, the photocatalytic performance is diminished.

In summary, a comprehensive understanding is drawn: the coupling of ZnTiO_3_ and TiO_2_ creates semiconductor junctions, enhancing charge generation under light irradiation. However, the coupling simultaneously induces a decrease in crystallinity and an increase in amorphous composition, both of which hinder the migration of photogenerated charges. This dual effect leads to the creation of additional photogenerated charge recombination centers, increasing recombination rates and consequently undermining photocatalytic activity.

## 3. Materials and Methods

### 3.1. Materials

Anhydrous ethanol (C_2_H_5_OH, AR, ≥99.7%), tetrabutyl titanate (Analytical Reagent, AR, ≥98.0%), glacial acetic acid (C_2_H_4_O_2_, AR, ≥99.5%), zinc nitrate hexahydrate (Zn(NO_3_)_2_·6(H_2_O), AR) were purchased from Chengdu Kelong Chemical Co., Ltd., Chengdu, China.

### 3.2. Sample Preparation

60 mL anhydrous ethanol (CH_3_CH_2_OH) and 20 mL tetrabutyl titanate (C_16_H_36_O_4_Ti) were mixed and stirred for 30 min to gain solution A. 10 mL deionized water and 15 mL glacial acetic acid (C_2_H_4_O_2_) were mixed to obtain solution B, which was dropped into solution A to form sol. The obtained sol was aged for 24 h to obtain gel. After drying at 120 °C for 6 h, the gel was calcinated at 470 °C, 570 °C, and 670 °C for 1 h. The prepared samples were marked as PT-470, PT-570, and PT-670.

By adding 8.75 g zinc nitrate hexahydrate (Zn(NO_3_)_2_·6H_2_O) into B solution and keeping other steps the same as before, ZnTiO_3_/TiO_2_ composites with a molar ratio of Zn/Ti of 1:2 can be prepared, which were marked as ZT-470, ZT-570, and ZT-670.

### 3.3. Sample Characterization

The analysis of the crystal structure of the samples was conducted using a DX-2700 X-ray diffractometer (XRD) from Dandong Haoyuan Instrument Co. Ltd., located in Dandong, China. The morphology was assessed through a Hitachi SU8220 scanning electron microscope (SEM) and a JEM-F200 transmission electron microscope (TEM and HRTEM) from FEI Company, situated in Hillsboro, OR, USA. The specific surface area measurement was performed using an ASAP2460 analyzer based on the Brunauer-Emmett-Teller (BET) method. The chemical valence analysis was undertaken employing an XSAM800 multifunctional surface analysis system (XPS) from Thermo Scientific K-Alpha, provided by Kratos Ltd., Manchester, UK. For the investigation of photoluminescence (PL) spectra, an F-4600 fluorescence spectrum analyzer from the Shimadzu Group Company in Kyoto, Japan, was employed. The optical absorption characteristics were probed using a UV-3600 UV-visible spectrophotometer (DRS) from the same Shimadzu Group Company. Furthermore, the assessment of photocurrent curves (PC) and electrochemical impedance spectroscopy (EIS) was carried out using a DH-7000 electrochemical workstation, supplied by Jiangsu Donghua Analytical Instrument Co., Ltd. based in Jingjiang, China. Weigh 40 mg sample and 5 mg polyvinylidene fluoride, add 0.5 mL LN-methylpyrrolidone (DMF) to the mixture to obtain a uniform slurry, evenly coat on the pre-treated FTO plate, and dry at 120 °C s for 2 h to make the working electrode. Pt electrode, Ag/AgCl electrode, and 0.1 mol/L Na_2_SO_4_ as a counter electrode, reference electrode, and electrolyte, respectively. 200 W LED lights simulate sunlight as an external light source.

### 3.4. Photocatalysis Experiment

The assessment of the photocatalytic efficiency of the samples involved the degradation of an aqueous solution of methylene blue (MB) under the illumination of a 250 W xenon lamp. In this method, 0.10 g of the powdered sample was introduced into a 100 mL solution of MB with a concentration of 10 mg/L. At intervals of 15 min, aliquots of the solution were extracted for analysis. After undergoing centrifugation, the absorbance of the solution at 664 nm was quantified. The degradation extent of MB was calculated employing the following formula:

Degradation (%) = ((A_0_ − A_t_)/A_0_) × 100%

Here, A_0_ stands for the initial absorbance, and A_t_ denotes the absorbance at the specific time t. This method effectively quantifies the degradation percentage of MB, offering a tangible metric to gauge the photocatalytic effectiveness of the samples.

## 4. Conclusions

We synthesized two distinct photocatalysts, pure TiO_2_, and ZnTiO_3_/TiO_2_ composites, using the sol–gel method. Our investigation focused on the influence of calcination temperature on their microstructure and photocatalytic performance. The transition from anatase to rutile occurs at 570 to 670 °C, this pivotal shift engenders a heterophase configuration, wherein both anatase and rutile coexist. The mixed crystal structure significantly enhances the separation of carriers, resulting in PT-670 being the most photocatalytic activity. Within 60 min, the degradation degree of MB reaches 91%. The coupling with ZnTiO_3_ demonstrates a restraining effect on the phase transformation process. In this context, ZnTiO_3_/TiO_2_ synthesized at 670 °C was still anatase. Photocurrent tests showed that coupling with ZnTiO_3_ led to significantly higher carrier generation. However, these findings become more nuanced when we consider the outcomes of EIS and PL spectra. The decrease in crystallinity within the composite led to an increase in charge transfer resistance and a simultaneous rise in charge recombination. The coupling with ZnTiO_3_ inhibited the phase transformation, impeded charge transfer efficiency, and increased recombination, ultimately diminishing photocatalytic performance.

## Figures and Tables

**Table 1:** Average crystallization sizes and mass content of TiO_2_ samples.

Sample	2θ	FWHM	D (nm)
PT-470	25.3°	0.565	14.3 nm
PT-570	25.4°	0.387	20.8 nm
PT-670 Anatase (56.3%)	25.3°	0.228	35.3 nm
PT-670 Rutile (43.7%)	27.4°	0.150	53.6 nm
ZT-470	25.3°	0.863	9.3 nm
ZT-570	25.3°	0.724	11.1 nm
ZT-670	25.4°	0.298	27.0 nm

**Table 2:** Specific surface areas of samples.

Sample	Specific Surface Area
PT-470	54.5 m^2^/g
PT-570	12.5 m^2^/g
PT-670	1.3 m^2^/g
ZT-670	10.6 m^2^/g

**Table 3:** Band gaps of samples.

Sample	470 °C	570 °C	670 °C
PT	3.20 eV	3.22 eV	3.06 eV
ZT	3.21 eV	3.21 eV	3.23 eV

**Table 4:** First-order reaction rate constant k and R^2^.

Sample	k/min^−1^	R^2^
PT-470	0.025	0.997
PT-570	0.010	0.990
PT-670	0.039	0.995
ZT-470	0.007	0.991
ZT-570	0.008	0.999
ZT-670	0.008	0.996

**Table 5:** MB degradation degrees by various photocatalysts reported in the literature.

Photocatalyst	C_atalyst_ g·L^−1^	C_MB_ mg·L^−1^	Light Source	Decolorization Degree	Ref.
TiO_2_/g-C_3_N_5_	0.2	20	Xenon lamp (500 W)	97.4% in 180 min	[52]
Au/TiO_2_	0.5	10	Xenon lamp (300 W)	69.7% in 240 min	[53]
SnO_2_/TiO_2_	0.05	10	Halogen lamp (400 W)	100% in 180 min	[31]
TiO_2_/FeS_2_	1	25	Cool white lamp (104 W)	100% in 180 min	[54]
ZnO-TiO_2_	0.04	10	UV lamp (18 W)	59.6% in 120 min	[55]
PT-670	1	10	Xenon lamp (250 W)	91.0% in 60 min	Present work

## Data Availability

Data are contained within the article.
